# 6,10,16,19-Tetra­oxatrispiro­[4.2.2.4.2.2]nona­deca­ne

**DOI:** 10.1107/S1600536808001785

**Published:** 2008-01-23

**Authors:** Ji-Kui Wang, Hai-Bo Wang, Cong-Ren Wu, Jin-Tang Wang

**Affiliations:** a Department of Applied Chemistry, College of Science, Nanjing University of Technology, Nanjing 210009, People’s Republic of China

## Abstract

The asymmetric unit of the title compound, C_15_H_24_O_4_, contains one half-mol­ecule; a twofold rotation axis passes through the central C atom. The non-planar six- and five-membered rings adopt chair and envelope conformations, respectively. In the crystal structure, inter­molecular C—H⋯O hydrogen bonds link the mol­ecules.

## Related literature

For general background, see: Jermy & Pandurangan (2005 ▶). For related literature, see: Sun *et al.* (2001 ▶). For ring conformation puckering parameters, see: Cremer & Pople (1975 ▶). For bond-length data, see: Allen *et al.* (1987 ▶).
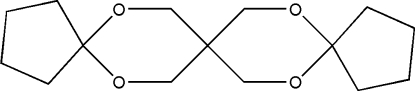


## Experimental

### 

#### Crystal data

C_15_H_24_O_4_
*M* *_r_* = 268.34
Monoclinic, 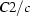

*a* = 25.605 (5) Å
*b* = 5.5820 (11) Å
*c* = 10.337 (2) Å
β = 90.22 (3)°
*V* = 1477.4 (5) Å^3^
*Z* = 4
Mo *K*α radiation
μ = 0.09 mm^−1^
*T* = 294 (2) K
0.30 × 0.20 × 0.10 mm

#### Data collection

Enraf–Nonius CAD-4 diffractometer
Absorption correction: ψ scan (North *et al.*, 1968 ▶) *T*_min_ = 0.965, *T*_max_ = 0.982
1547 measured reflections
1457 independent reflections
864 reflections with *I* > 2σ(*I*)
*R*_int_ = 0.050
3 standard reflections frequency: 120 min intensity decay: none

#### Refinement

*R*[*F* ^2^ > 2σ(*F* ^2^)] = 0.067
*wR*(*F* ^2^) = 0.173
*S* = 0.93
1457 reflections
87 parameters
H-atom parameters constrained
Δρ_max_ = 0.26 e Å^−3^
Δρ_min_ = −0.21 e Å^−3^

### 

Data collection: *CAD-4 Software* (Enraf–Nonius, 1989 ▶); cell refinement: *CAD-4 Software*; data reduction: *XCAD4* (Harms & Wocadlo, 1995 ▶); program(s) used to solve structure: *SHELXS97* (Sheldrick, 2008 ▶); program(s) used to refine structure: *SHELXL97* (Sheldrick, 2008 ▶); molecular graphics: *SHELXTL* (Sheldrick, 2008 ▶); software used to prepare material for publication: *PLATON* (Spek, 2003 ▶).

## Supplementary Material

Crystal structure: contains datablocks global, I. DOI: 10.1107/S1600536808001785/hk2414sup1.cif

Structure factors: contains datablocks I. DOI: 10.1107/S1600536808001785/hk2414Isup2.hkl

Additional supplementary materials:  crystallographic information; 3D view; checkCIF report

## Figures and Tables

**Table 1:** Hydrogen-bond geometry (Å, °)

*D*—H⋯*A*	*D*—H	H⋯*A*	*D*⋯*A*	*D*—H⋯*A*
C6—H6*B*⋯O2^i^	0.97	2.58	3.413 (4)	143

Symmetry code: (i) 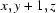.

**Fig. 1.**
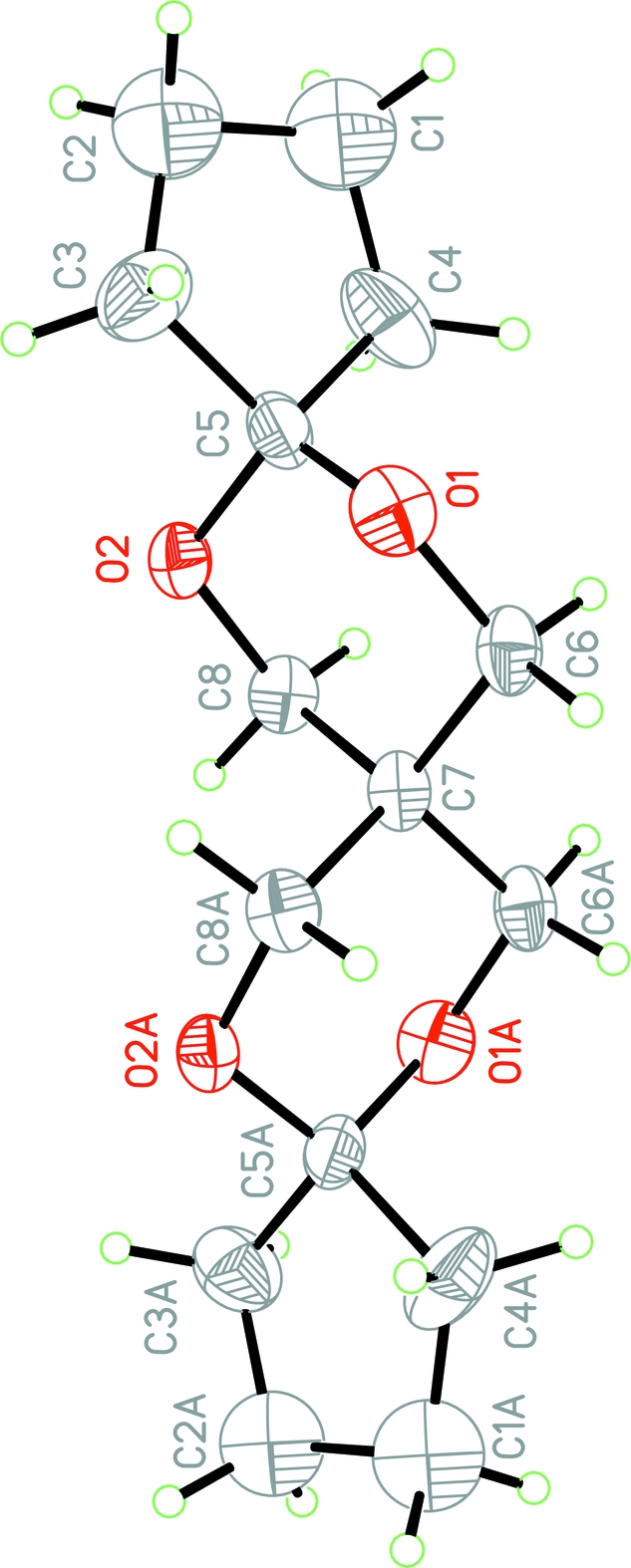
The molecular structure of the title molecule, showing the atom-numbering scheme. Displacement ellipsoids are drawn at the 50% probability level. [Symmetry code: (A) 1 - x, y, 1/2 - z.]

**Fig. 2.**
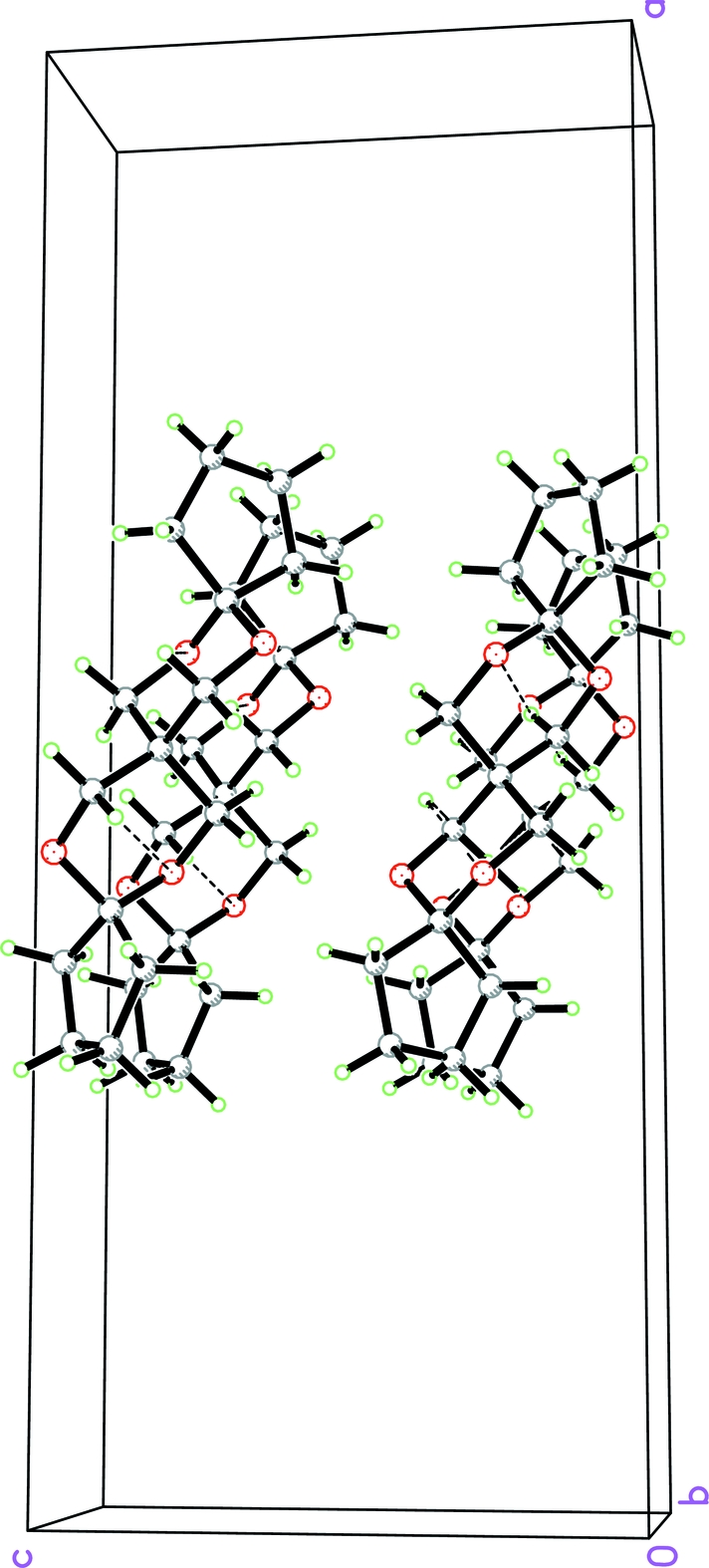
A packing diagram of (I). Hydrogen bonds are shown as dashed lines.

## supplementary crystallographic information

### Comment

The title compound, (I), is an important intermediate in the synthesis of pesticides (Jermy & Pandurangan, 2005). The crystal structure determination of (I) has been carried out in order to elucidate the molecular conformation.

The asymmetric unit of the title compound, (I), contains one-half molecule (Fig. 1), in which the bond lengths are within normal ranges (Allen *et al.*, 1987).

Ring B (O1/O2/C5—C8) is not planar, having total puckering amplitude, Q_T_, of 0.943 (3) Å. It adopts chair conformation [φ = -32.96 (2)° and θ = 58.52 (3)°] (Cremer & Pople, 1975). Ring A has envelope conformation with atom C1 displaced by -0.222 (3) Å from the plane of the other ring atoms.

In the crystal structure, intermolecular C—H···O hydrogen bonds (Table 1) link the molecules, in which they may be effective in the stabilization of the structure.

### Experimental

The title compound was prepared from a mixture of 2,2-bis-(hydroxymethyl) propane-1,3-diol (0.68 g, 5 mmol), cyclopentanone (10 mmol), freshly activated catalyst TiO2/SO4(2-)(0.6 g, 0.32 mmol) and cyclohexane (80 ml), heated with stirring at refluxing temperature for 2 h, using a Dean-Stark apparatus in a nitrogen atmosphere (Sun *et al.*, 2001). The progress of the reaction was monitored by thin-layer chromatography. After cooling to room temperature, the catalyst was filtered off, the crude product was isolated by distillation and the solid was recrystallized from ethanol. Crystals of (I) were obtained by dissolving the title compound (1.0 g) in toluene (15 ml) and evaporating the solvent slowly at room temperature for about 7 d.

### Refinement

H atoms were positioned geometrically, with C—H = 0.97 Å for methylene H, and constrained to ride on their parent atoms, with *U*_iso_(H) = 1.2*U*_eq_(C).

### Crystal data

**Table:**

C_15_H_24_O_4_	*F*_000_ = 584
*M**_r_* = 268.34	*D*_x_ = 1.206 Mg m^−^^3^
Monoclinic, *C*2/*c*	Melting point: 401 K
Hall symbol: -C 2yc	Mo *K*α radiation λ = 0.71073 Å
*a* = 25.605 (5) Å	Cell parameters from 25 reflections
*b* = 5.5820 (11) Å	θ = 10–13º
*c* = 10.337 (2) Å	µ = 0.09 mm^−^^1^
β = 90.22 (3)º	*T* = 294 (2) K
*V* = 1477.4 (5) Å^3^	Block, colorless
*Z* = 4	0.30 × 0.20 × 0.10 mm

### Data collection

**Table:**

Enraf–Nonius CAD-4 diffractometer	*R*_int_ = 0.050
Radiation source: fine-focus sealed tube	θ_max_ = 26.0º
Monochromator: graphite	θ_min_ = 1.6º
*T* = 294(2) K	*h* = −31→31
ω/2θ scans	*k* = 0→6
Absorption correction: ψ scan(North *et al.*, 1968)	*l* = 0→12
*T*_min_ = 0.965, *T*_max_ = 0.982	3 standard reflections
1547 measured reflections	every 120 min
1457 independent reflections	intensity decay: none
864 reflections with *I* > 2σ(*I*)	

### Refinement

**Table:**

Refinement on *F*^2^	Secondary atom site location: difference Fourier map
Least-squares matrix: full	Hydrogen site location: inferred from neighbouring sites
*R*[*F*^2^ > 2σ(*F*^2^)] = 0.067	H-atom parameters constrained
*wR*(*F*^2^) = 0.173	*w* = 1/[σ^2^(*F*_o_^2^) + (0.06*P*)^2^ + 4.5*P*] where *P* = (*F*_o_^2^ + 2*F*_c_^2^)/3
*S* = 0.93	(Δ/σ)_max_ < 0.001
1457 reflections	Δρ_max_ = 0.26 e Å^−^^3^
87 parameters	Δρ_min_ = −0.21 e Å^−^^3^
Primary atom site location: structure-invariant direct methods	Extinction correction: none

### Special details

**Table:**

Geometry. All e.s.d.'s (except the e.s.d. in the dihedral angle between two l.s. planes) are estimated using the full covariance matrix. The cell e.s.d.'s are taken into account individually in the estimation of e.s.d.'s in distances, angles and torsion angles; correlations between e.s.d.'s in cell parameters are only used when they are defined by crystal symmetry. An approximate (isotropic) treatment of cell e.s.d.'s is used for estimating e.s.d.'s involving l.s. planes.
Refinement. Refinement of *F*^2^ against ALL reflections. The weighted *R*-factor *wR* and goodness of fit *S* are based on *F*^2^, conventional *R*-factors *R* are based on *F*, with *F* set to zero for negative *F*^2^. The threshold expression of *F*^2^ > σ(*F*^2^) is used only for calculating *R*-factors(gt) *etc*. and is not relevant to the choice of reflections for refinement. *R*-factors based on *F*^2^ are statistically about twice as large as those based on *F*, and *R*- factors based on ALL data will be even larger.

### Fractional atomic coordinates and isotropic or equivalent isotropic displacement parameters (Å^2^)

**Table:**

	*x*	*y*	*z*	*U*_iso_*/*U*_eq_	
O1	0.43380 (7)	0.3857 (4)	0.41870 (16)	0.0381 (5)	
O2	0.42692 (7)	0.0861 (3)	0.26166 (16)	0.0324 (5)	
C1	0.30826 (17)	0.3438 (9)	0.3294 (5)	0.0889 (15)	
H1A	0.2819	0.3125	0.2638	0.107*	
H1B	0.2974	0.4816	0.3797	0.107*	
C2	0.31481 (15)	0.1237 (9)	0.4179 (4)	0.0803 (13)	
H2A	0.2973	0.1514	0.4996	0.096*	
H2B	0.2993	−0.0159	0.3773	0.096*	
C3	0.36848 (12)	0.0842 (7)	0.4393 (3)	0.0503 (9)	
H3A	0.3768	−0.0835	0.4262	0.060*	
H3B	0.3777	0.1271	0.5274	0.060*	
C4	0.35773 (13)	0.3897 (7)	0.2697 (3)	0.0567 (10)	
H4A	0.3662	0.5588	0.2750	0.068*	
H4B	0.3567	0.3436	0.1792	0.068*	
C5	0.39900 (11)	0.2397 (5)	0.3435 (3)	0.0336 (7)	
C6	0.46853 (11)	0.5234 (5)	0.3396 (2)	0.0342 (7)	
H6A	0.4922	0.6138	0.3945	0.041*	
H6B	0.4484	0.6366	0.2884	0.041*	
C7	0.5000	0.3637 (7)	0.2500	0.0278 (8)	
C8	0.46173 (10)	0.2072 (5)	0.1738 (2)	0.0337 (7)	
H8A	0.4417	0.3060	0.1145	0.040*	
H8B	0.4809	0.0901	0.1235	0.040*	

### Atomic displacement parameters (Å^2^)

**Table:**

	*U*^11^	*U*^22^	*U*^33^	*U*^12^	*U*^13^	*U*^23^
O1	0.0461 (12)	0.0405 (12)	0.0275 (9)	0.0017 (10)	−0.0016 (8)	−0.0038 (9)
O2	0.0368 (10)	0.0236 (10)	0.0368 (10)	−0.0008 (9)	0.0005 (8)	−0.0038 (8)
C1	0.076 (3)	0.098 (4)	0.093 (3)	0.005 (3)	−0.001 (2)	0.016 (3)
C2	0.069 (3)	0.089 (3)	0.083 (3)	−0.005 (3)	0.006 (2)	0.009 (3)
C3	0.052 (2)	0.053 (2)	0.0460 (17)	−0.0121 (17)	0.0100 (15)	−0.0008 (16)
C4	0.057 (2)	0.052 (2)	0.061 (2)	0.0252 (18)	−0.0165 (17)	−0.0025 (17)
C5	0.0338 (14)	0.0256 (15)	0.0413 (15)	0.0057 (12)	−0.0035 (12)	−0.0032 (12)
C6	0.0469 (16)	0.0252 (15)	0.0306 (13)	−0.0021 (13)	−0.0012 (12)	−0.0044 (11)
C7	0.040 (2)	0.023 (2)	0.0204 (16)	0.000	−0.0036 (15)	0.000
C8	0.0393 (15)	0.0322 (16)	0.0296 (13)	−0.0017 (13)	−0.0019 (12)	−0.0004 (12)

### Geometric parameters (Å, °)

**Table:**

O1—C5	1.434 (3)	C3—H3B	0.9700
O1—C6	1.434 (3)	C4—C5	1.547 (4)
O2—C5	1.402 (3)	C4—H4A	0.9700
O2—C8	1.443 (3)	C4—H4B	0.9700
C1—C4	1.434 (5)	C6—C7	1.519 (3)
C1—C2	1.540 (6)	C6—H6A	0.9700
C1—H1A	0.9700	C6—H6B	0.9700
C1—H1B	0.9700	C7—C6^i^	1.519 (3)
C2—C3	1.408 (5)	C7—C8	1.529 (3)
C2—H2A	0.9700	C7—C8^i^	1.529 (3)
C2—H2B	0.9700	C8—H8A	0.9700
C3—C5	1.533 (4)	C8—H8B	0.9700
C3—H3A	0.9700		
			
C5—O1—C6	112.37 (19)	H4A—C4—H4B	108.6
C5—O2—C8	114.2 (2)	O2—C5—O1	110.9 (2)
C4—C1—C2	107.7 (4)	O2—C5—C3	107.8 (2)
C4—C1—H1A	110.2	O1—C5—C3	106.8 (2)
C2—C1—H1A	110.2	O2—C5—C4	112.5 (2)
C4—C1—H1B	110.2	O1—C5—C4	112.4 (2)
C2—C1—H1B	110.2	C3—C5—C4	106.0 (3)
H1A—C1—H1B	108.5	O1—C6—C7	111.4 (2)
C3—C2—C1	108.8 (4)	O1—C6—H6A	109.4
C3—C2—H2A	109.9	C7—C6—H6A	109.4
C1—C2—H2A	109.9	O1—C6—H6B	109.4
C3—C2—H2B	109.9	C7—C6—H6B	109.4
C1—C2—H2B	109.9	H6A—C6—H6B	108.0
H2A—C2—H2B	108.3	C6—C7—C6^i^	108.1 (3)
C2—C3—C5	108.0 (3)	C6—C7—C8	107.99 (15)
C2—C3—H3A	110.1	C6^i^—C7—C8	111.22 (14)
C5—C3—H3A	110.1	C6—C7—C8^i^	111.22 (14)
C2—C3—H3B	110.1	C6^i^—C7—C8^i^	107.99 (15)
C5—C3—H3B	110.1	C8—C7—C8^i^	110.3 (3)
H3A—C3—H3B	108.4	O2—C8—C7	109.86 (18)
C1—C4—C5	107.1 (3)	O2—C8—H8A	109.7
C1—C4—H4A	110.3	C7—C8—H8A	109.7
C5—C4—H4A	110.3	O2—C8—H8B	109.7
C1—C4—H4B	110.3	C7—C8—H8B	109.7
C5—C4—H4B	110.3	H8A—C8—H8B	108.2

Symmetry codes: (i) −*x*+1, *y*, −*z*+1/2.

### Hydrogen-bond geometry (Å, °)

**Table:**

*D*—H···*A*	*D*—H	H···*A*	*D*···*A*	*D*—H···*A*
C6—H6B···O2^ii^	0.97	2.58	3.413 (4)	143

Symmetry codes: (ii) *x*, *y*+1, *z*.
